# Ciprofloxacin-Loaded Silver Nanoparticles as Potent Nano-Antibiotics against Resistant Pathogenic Bacteria

**DOI:** 10.3390/nano12162808

**Published:** 2022-08-16

**Authors:** Duaa R. Ibraheem, Nehia N. Hussein, Ghassan M. Sulaiman, Hamdoon A. Mohammed, Riaz A. Khan, Osamah Al Rugaie

**Affiliations:** 1 Division of Biotechnology, Department of Applied Sciences, University of Technology, Baghdad 10066, Iraq; 2 Department of Medicinal Chemistry and Pharmacognosy, College of Pharmacy, Qassim University, Buraidah 51452, Saudi Arabia; 3 Department of Pharmacognosy and Medicinal Plants, Faculty of Pharmacy, Al-Azhar University, Cairo 11371, Egypt; 4 Department of Basic Medical Sciences, College of Medicine and Medical Sciences, Qassim University, Unaizah 51911, Saudi Arabia

**Keywords:** ciprofloxacin, PEG, silver nanoparticles, nanocomposite, antioxidant, pathogenic bacteria

## Abstract

Silver nanoparticles (AgNPs) have demonstrated numerous physicochemical, biological, and functional properties suitable for biomedical applications, including antibacterial and drug carrier properties. In the present study, the antibiotic, ciprofloxacin (CIP), was loaded onto AgNPs, which were synthesized via the chemical reduction method, thereby enhancing CIP’s antibacterial activity against Gram-negative (*Acinetobacter baumannii* and *Serratia marcescens*) and Gram-positive (*Staphylococcus aureus*) bacterial strains. Polyethylene glycol–400 (PEG) was used to prepare an AgNPs-PEG conjugate with enhanced stability and to act as the linker between CIP and AgNPs, to produce the novel nanocomposite, AgNPs-PEG-CIP. The prepared AgNPs and their conjugates were characterized by ultraviolet-visible spectrophotometry, Fourier-transform infrared spectroscopy, X-ray diffraction, field emission scanning electron microscopy with energy-dispersive X-ray spectroscopy, transmission electron microscopy, zeta potential analysis, and dynamic light scattering techniques. The inhibitory activity of AgNPs and their conjugates on the growths of pathogenic bacteria was assessed using the well-diffusion method. The results showed the enhanced antibacterial effects of AgNPs-CIP compared to CIP alone. The AgNPs-PEG-CIP nanocomposite showed excellent inhibitory effects against bacterial isolates, with its inhibition zones diameters reaching 39, 36, and 40 mm in *S. aureus*, *A. baumannii*, and *S. marcescens*, respectively. The minimum inhibitory concentration and minimum bactericidal concentration of fogNPs and their conjugates and their antibiofilm effects were also determined. The antioxidant potentials of AgNPs and their conjugates, tested via their 1,1-diphenyl-2-picryl-hydrazyl (DPPH) scavenging ability, showed that the activity increased with increasing AgNPs concentration and the addition of the PEG and/or CIP. Overall, according to the results obtained in the present study, the new nanocomposite, AgNPs-PEG-CIP, showed the highest antibacterial, antibiofilm, and antioxidant activity against the pathogenic bacteria tested, compared to CIP alone. The preparation has high clinical potential for prospective use as an antibacterial agent.

## 1. Introduction

In recent decades, the increasing resistance of microorganisms to antibiotics has resulted in serious health issues, and it is still a major concern worldwide regarding the use of the majority of antibiotics [1]. The frequent use of antibiotics has led to microorganism resistance, which was first characterized by the development of microorganisms’ resistance to treatment, decreased therapeutic indices, toxicity, side effects, non-specific effects, and difficulties in dosage [2]. Alongside the increase in antibiotic resistance over the past decade, mostly because of the widespread and incorrect use of these therapeutic agents, the need for better-acting antibiotics has been continuously sought throughout the resistance period. With the advent of the discovery of silver nanoparticles (AgNPs) as an effective anti-bacterial agent, investigations into the possible effectiveness of AgNPs for treating pathogenic infections have been ongoing. In this context, nanotechnology, an important tool in nanomedicine development, has provided successful solutions to the aforementioned problems in terms of the feasibility of manufacturing particles measuring between 1 and 100 nm in size, with unique physical and chemical properties. The small size and the increased surface area-to-volume ratio of the particles enable them to function as delivery vehicles for several therapeutic agents in different organs of the body. The nanoparticles (NPs) provide high drug-loading capacity with an increased half-life, transitional stability, and safety from toxicity, site-directed targeted delivery, and the controlled release of the therapeutic agent(s), together with increased levels of antibacterial activity owing to high loading, increased bioavailability, lesser toxicity, and the enhanced half-life of the therapeutic agent [3]. The metal-NPs (MNPs) are among the most extensively used nanomaterials due to their pronounced antibacterial properties [4]. Two theories have been offered to explain the antibacterial properties of MNPs: (i) the dissolution of metals from the MNPs, which causes metal ion toxicity inside the cells, and (ii) the formation of reactive oxygen species (ROS) on MNPs, thereby causing oxidative stress inside the cells, thereby triggering cell death [5]. The successful use of various nanoparticle- and nanotechnology-based therapies against pathogenic microorganisms have greatly contributed to the development of the advanced genre of nanomedicines [6].

Silver NPs (AgNPs) are the most common types of nano-scale antibiotics inhibiting bacterial growth. The AgNPs are chemically stable, inert, present high binding capacity for the incoming therapeutic entity, are stable and safe in the biological environment, and also offer low toxicity [7]. The AgNPs hybrids have also been reported to respond to temperature [8]. AgNPs have also been reported to be among the most important antibacterial, antifungal, and antiviral agents that have been widely utilized in surface modification strategies for development as a drug delivery agent in biomedical applications [9], due to their ability to conjugate with the sulfur in sulfhydryl and other sulfur-based function groups that are found in bacterial respiratory enzymes. The AgNPs also bind phosphorous-containing components, such as deoxyribonucleic acid (DNA), and thus prevent its duplication process. They also inactivate essential proteins and other cell functions, as well as inhibit the antioxidant system taking care of the free radicals to eventually inflict non-recoverable damage to the cells. Additionally, as antioxidants, the AgNPs are well known for their capacity to scavenge free radicals and protect the body against diseases, aging, and cancers, which are caused by the generated oxidative stress caused by ROS. ROS reactivity produces free radicals and other sub-molecular entities, such as hydroxyl radicals, hydrogen peroxide, and singlet oxygen; these are major factors in the progression of many physiological and biochemical malfunctions and are the cause of certain diseases, including infections and other adverse health outcomes. AgNPs have been found to be immensely useful in disease control and treatment because of their antioxidant properties [10,11,12]. Recently, the coined term “Nanobiotics” appeared in medical science as an alternative to “nano-scale antibiotics”. Researchers at Hangbang University in Seoul have combined AgNPs with certain antibiotics; these AgNPs have been shown to kill about 650 disease-causing microorganisms without harming the human body [6]. This combination has allowed the solving of different issues associated with bacterial resistance to antibiotics. Their antibiotic activity against several types of pathogens after conjugation with different NPs has also been shown to improve because of their increased permeability, caused by the weakening of the cellular membranes [5,11]. For example, when tested against *Escherichia coli* and *Staphylococcus aureus*, rifampicin and tetracycline hydrochloride-functionalized iron oxide NP-based formulations showed strong antibacterial effects [13].

In the course of the present study, ciprofloxacin (CIP) was conjugated with chemically synthesized AgNPs to prepare a new nano-based composite with improved antibacterial activity against three bacteria (*S. aureus*, *Acinetobacter baumannii*, and *S. marcescens*). CIP is a second-generation fluoroquinolone and is among one of the most commonly prescribed antibiotics owing to its broad bacterial inhibitory spectrum against the pathogens responsible for a number of organ infections, including urinary, intestinal, respiratory, skin, and soft-tissue infections, as well as sexually transmitted infections [14]. CIP is also active against several bacterial strains that are responsible for community-acquired pneumonia, and *Escherichia coli*, *Haemophilus influenzae*, *Klebsiella pneumoniae*, *Legionella pneumophila*, *Moraxella catarrhalis*, *Proteus mirabilis*, *Pseudomonas aeruginosa*, along with methicillin-sensitive *Staphylococcus aureus*, *Streptococcus pneumoniae*, and *Enterococcus faecalis*, compared to other new fluoroquinolones. Nonetheless, CIP is now the most frequently prescribed antibiotic for adult patients, although it has also shown resistance to *Streptococcus pyogenes*, *Klebsiella pneumonia*, *Burkholderia cepacia*, *Clostridium innocuum*, and *Enterococcus faecium* to certain degrees. Because it has shown inhibitory effects on both Gram-positive and Gram-negative bacteria, CIP is also among the most widely used antibiotics [15], and therefore was chosen to demonstrate the effectiveness of the prepared nanobiotics to overcome issues related to dose control, adverse effects, biological half-life (free CIP remains for 4–6 h in serum), and bioavailability at the site of action. CIP targets the DNA gyrase topoisomerase II and IV, thereby inhibiting the DNA replication process. While the activity of CIP against *S. aureus* results from topoisomerase IV targeting, its activity against Gram-negative bacteria is the result of DNA gyrase targeting [16].

The AgNPs-CIP conjugates stand out among the MNP-antibiotic conjugates because both the components, CIP and AgNPs, exhibit strong antibacterial properties through different processes, and both are used at relatively low doses, which also reduces the AgNPs-CIP toxicity [17]. The chelation and interaction of AgNPs with CIP are thought to result in structures comprising an Ag metal nano-core, surrounded by the antibiotic molecules, and such conjugates have been suggested to promote CIP persistence and continuous release at the site of bacterial contact, thus potentially facilitating the antibiotic’s increased bioavailability and, therefore, its perceived enhanced bioactivity [18].

Several natural and synthetic-origin polymers, such as polyethylene glycol (PEG), have been employed to generate coated AgNPs (e.g., AgNPs-PEG) as prospective safe formulations to sustain the increased duration of biological activity, offering enhanced biological half-life, increased transitional stability, and low toxicity [19]. Due to their improved mechanical qualities, non-toxicity, non-carcinogenic nature, and non-immunogenic effects, together with biocompatibility, bio-adhesive properties, and enhanced water solubility, PEGs have been employed as a matrix for delivering non-agglomerated AgNPs [20,21]. This non-ionic polymer is commonly used in drug conjugates; solid-sphere NPs (SNPs) (usually metal-based) functionalized PEGs have also shown enhanced biocompatibility. The PEG acts as the intermediate entity and also as a molecular spacer and a linker for CIP attachments onto the surfaces of SNPs, i.e., AgNPs, to improve drug loading and its subsequent release [22]. Therefore, AgNPs-PEG-CIP nano-based composites, owing to their biocompatibility and enhanced drug loading, together with other physicochemical characteristics, seem a viable option that may have strong antibacterial effects compared to the nanobiotics that have previously been prepared.

The primary aim of the present study was to improve the antibacterial activity of CIP by synthesizing CIP-loaded AgNPs through the intermediary PEG acting as the linker, for which the PEG is considered to provide a higher density of attachment sites for CIP loading on the AgNPs-PEGs’ surfaces to produce nano-structured AgNPs-PEG-CIP as the final formulation of the intended nanomedicine. The role of PEGs as structural units to inhibit NP aggregation during synthesis, to avoid the resulting agglomeration and entanglements with other nano-structures (both ready and residual) in the formulation colloid, is deemed to be crucial, although the role of PEGs in inhibiting the agglomeration and aggregate-forming behavior of the AgNPs was unsatisfactory, as demonstrated by the visual analysis of the samples; nonetheless, it worked to some extent and yielded active nano-colloidal suspensions. The PEGs’ covering of the AgNPs also provided stability, a safety coating for the reactive, bare AgNPs, provided zeta potential control, and improved polydispersity index for the size distribution of the prepared nano-formulation, an important factor in size distribution, contributing to the drug’s loading and release. The physicochemical properties of size, shape, size distribution, XRD-based composition, EDX analyses, and the antibacterial, antioxidant, and antibiofilm properties of the bare AgNPs, AgNPs-PEG, and AgNPs-PEG-CIP were investigated and compared to understand the roles and the reactivity and biological actions of these different nano-entities. A chemical interaction outlook between the AgNPs-PEG and CIP was achieved with an in silico model to indicate the intricacies of the chemical structures and the interactive points of the CIP structures. To the best of our knowledge, this is the first study investigating the antibacterial effect of AgNPs-PEG-CIP against selected multidrug-resistant bacteria on a model scale, such as *S. aureus*, *A. baumannii*, and *S. marcescens*.

## 2. Materials and Methods

### 2.1. Materials and Reagents

The chemicals and reagents used to synthesize AgNPs, AgNPs-PEG, AgNPs-CIP, and AgNPs-PEG-CIP were: silver nitrate (AgNO_3_, molecular weight (M_w_): 169.87 g moL^−1^), purchased from BDH, West Yorkshire, England; tri-sodium citrate di-hydrate (Na_3_C_6_H_5_O_7_, M_w_: 294.10 g moL^−1^) and sodium dodecyl sulfate (SDS, C_12_H_25_NaSO_4_, M_w_: 288.38 g moL^−1^), purchased from CDH, New Delhi, India; PEG (~98% purity, M_w_: 400 g moL^−1^), purchased from HiMedia, Thane West, India; deionized water, which was used as a solvent, obtained from Baghdad Company, Baghdad, Iraq; and CIP (M_w_: 367.9 g moL^−1^, high purity, ~98%), bought from Bio Basic, Markham ON, Canada. The media and reagents for antibacterial applications were: brain-heart infusion broth, obtained from Biolab, Samut Prakan, Thailand; Muller-Hinton agar medium, obtained from Mast, Liverpool, England; tryptic soy broth (TSB), phosphate buffer saline (PBS), and crystal violet (CV) provided by BDH, West Yorkshire, England; and 1,1-diphenyl-2-picryl-hydrazyl (DPPH, M_w_: 394.32 g moL^−1^), provided by CDH, New Delhi, India.

### 2.2. Bacterial Isolates

The pathogenic Gram-negative bacterial isolates, *A. baumannii* and *S. marcescens*, and the Gram-positive bacterium isolate, *S. aureus*, were obtained from the microbiology laboratory of the Medical City Hospital, Baghdad, Iraq. Isolates were identified using the VITEK system (VITEK, Biomérieux, Marcy-l’Etoile, France), kindly supplied by the Division of Biotechnology in the Department of Applied Science, University of Technology, Baghdad, Iraq.

### 2.3. Synthesis of Silver Nanoparticles and Their Combinations

#### 2.3.1. Synthesis of Bare AgNPs and Coated AgNPs-PEG

AgNPs-PEG were prepared according to Turkevich′s method [22], with some modifications. Trisodium citrate dihydrate was used as a reducing agent, with SDS as a capping agent. AgNO_3_ (4 Mm) was first dissolved in 100 mL of deionized water on a hot plate (80 °C) with magnetic stirring. Then, a mixture of trisodium citrate dehydrate (0.4 Mm) and SDS (0.5 mM), also dissolved in 100 mL of deionized water, were dropped for 30 min under continuous stirring. The final mixture was kept at 80 °C and 350 rpm for 2 h. When the solution’s color changed to yellow, indicating AgNP formation, it was cooled to room temperature and then stored in the refrigerator in the dark. AgNPs-PEG were obtained as previously described [23]. Briefly, an aqueous solution of PEG-400 was added to synthesized bare AgNPs at a ratio of 1:1 under continuous stirring for 12 h to obtain colloidal AgNPs-PEG.

#### 2.3.2. Preparation of AgNPs-CIP and AgNPs-PEG-CIP

AgNPs-CIP were prepared as previously described [24]. An aqueous solution of CIP (0.001 M) was added to 100 mL of the previously synthesized AgNPs, under ultra-sonication for 2 h at room temperature, to enhance the interaction between CIP and the AgNPs. When the solution changed color from yellow to orange, successful AgNPs-CIP conjugation was considered to have been achieved. The same procedure was used for preparing the AgNPs-PEG-CIP nanocomposite by adding an aqueous solution of CIP at 0.001 M to 20 mL of the prepared AgNPs-PEG.

### 2.4. Characterization of AgNPs and Their Conjugates

AgNPs, AgNPs-PEG, AgNPs-CIP, and AgNPs-PEG-CIP were characterized by chromatography analysis with a LAMBDA 365 spectrophotometer (PerkinElmer, Waltham, MA, USA) for the ultraviolet-visible (UV-Vis) spectrum, using continuous scanning (200–800 nm) to detect the surface Plasmon resonance (SPR) peaks of the formed NPs. X-ray diffraction (XRD) analysis was performed on a Powder XRD 2700AB diffractometer (HAOYUAN, Zhejiang, China) to confirm the crystal structure (crystal phases and crystallite size) of the prepared NPs. The crystallite size (D) of AgNPs and their conjugates were calculated using Scherrer’s equation:

D = Kλ/β cos θ(1)

where λ is the wavelength of the X-rays, β is the full width at half-maximum of the diffraction peak, and θ is the Bragg’s angle. In the present study, the system was supplied with copper-kα radiation at a wavelength (λ) of 1.5406 nm, produced at 40 kV. Fourier transform infrared spectroscopy (FT-IR, PerkinElmer, Waltham, MA, USA) was performed to detect the active groups present in the samples, within the 4000–500 cm^−1^ range and at 4 cm^−1^ resolution, identifying the molecular vibrations in the particles. Field-emission scanning electron microscopy (FE-SEM, Inspect F50-FE-SEM, FEI, Eindhoven, The Netherlands) was used to study the NPs size, shape, and surface morphology. Transmission electron microscopy (TEM, Zeiss, Jena, Germany) was used to identify the morphological features of NPs. The zeta potential and dynamic light scattering analysis of AgNPs and their conjugates were determined using a Zeta-Plus analyser (Zeta, Brookhaven, Deklab County, GA, USA) to determine the stability of the prepared NPs and their surface charge, as well as for evaluating the NP size distribution in solutions and colloidal suspensions.

### 2.5. Antioxidant Activity

The antioxidant activity of the NPs was evaluated as previously described in [25], with minor adjustments. Utilizing stable DPPH radicals, the scavenging activities of AgNPs, AgNPs-PEG, AgNPs-CIP, and AgNPs-PEG-CIP were evaluated at 6.25, 12.5, 25, 50, and 100 μg mL^−1^. Seven hundred and fifty microliters of each sample were mixed with 750 μL of previously prepared DPPH solution (0.02 g DPPH in 50 mL methanol). To prepare the negative control, 750 μL of DPPH solution was mixed with 750 μL of methanol, while the positive control was prepared by mixing 750 μL of DPPH with 0.5 g of ascorbic acid (vitamin C) at a concentration of 5 µg mL^−1^. The test samples and controls were kept at 37 °C for 30 min and were protected from light before determining their optical density (OD) at 517 nm. The sample with the lowest OD showed the highest scavenging activity (%) of DPPH, which was calculated using Equation (2):

(2) $$\mathrm{DPPH}\;\mathrm{scavenging}\;\mathrm{activity}\;=\frac{\left(\mathrm{OD}\;\mathrm{of}\;\mathrm{Control}-\mathrm{OD}\;\mathrm{of}\;\mathrm{Sample}\right)}{\mathrm{OD}\;\mathrm{of}\;\mathrm{Control}\;}\times 100$$

### 2.6. Antibacterial Activity

The synergistic effect of AgNPs, CIP, AgNPs-CIP, AgNPs-PEG, and AgNPs-PEG-CIP on the growth of bacterial isolates were evaluated using Mueller-Hinton agar medium and *S. aureus*, *A. baumannii*, and *S. marcescens* bacterial suspensions. The McFarland standard was used to obtain suspensions at 1.5 × 10^8^ colony-forming units (CFU) mL^−1^. Mueller–Hinton agar medium plates were swabbed with each bacterial suspension, then six wells at 8 mm diameter were punched out of each agar plate using a sterilized well cutter. The wells were loaded with 80 μL of AgNPs, CIP, AgNPs-CIP, AgNPs-PEG, or AgNPs-PEG-CIP for each tested bacterium. Deionized water was used as the control. After incubation at 37 °C for 24 h, the inhibition zones were observed around each well and measured (to the nearest mm) to determine the antibacterial activity of AgNPs, CIP, AgNPs-CIP, AgNPs-PEG, and AgNPs-PEG-CIP. The folds increase in the diameters of the inhibition zones after adding PEG-400 and for CIP after adding AgNPs were compared to the diameters of the inhibition zones when AgNPs and CIP alone were used, by applying Equation (3):

(3) Fold increase (FI)=[(b−a)/a]×100

where *a* represents the diameters of the inhibition zones of AgNPs or CIP alone, and *b* represents the diameters of the inhibition zones of the AgNPs-PEG and AgNPs-CIP [26].

### 2.7. Antibiofilm Effect

The biofilm inhibitory concentrations of AgNPs were determined using the tube method [27]. Briefly, a single colony of each bacterium was grown for 24 h in 5 mL of TSB medium in test tubes containing 1 mL of AgNPs, AgNPs-PEG, AgNPs-CIP, or AgNPs-PEG-CIP (at 12.5, 25, 50, or 100 μg mL^−1^ concentrations). After the incubation period, the medium was discarded and the tubes were washed with a PBS solution (pH 7.3), left to air-dry, and then stained with 0.1% CV for 5 min. The excess dye was washed off, and the tubes were left inverted until dried. If there was no biofilm formation, a (−) was recorded; (+), (++), or (+++) were attributed for weak, medium, or dense biofilm formations, respectively.

### 2.8. Determination of MIC and MBC

The MIC and MBC of the AgNPs and their PEG and/or CIP conjugates were calculated as previously described [28]. Briefly, 0.1 mL of AgNPs, AgNPs-PEG, AgNPs-CIP, and AgNPs-PEG-CIP at different concentrations (12.5, 25, 50, or 100 μg mL^−1^) were added with 0.1 mL of a *S. aureus*, *A. baumannii*, or *S. marcescens* suspension to test tubes containing 0.8 mL of BHI broth, and then compared to McFarland standard tubes. The tubes were vigorously shaken and incubated at 37 °C for 24 h before recording the turbidity. Then, 100 μL of each mixture was spread on Muller–Hinton agar medium plates and incubated for another 24 h at 37 °C. The results were recorded as the presence, or absence, of bacterial growth on the agar plates.

### 2.9. Statistical Analyses

The data were analyzed with a one-way analysis of variance (ANOVA) using SPSS v.24 software at a 0.05 level of statistical significance. The data were presented as mean ± SE. All experiments were carried out in triplicate.

## 3. Results and Discussion

### 3.1. Synthesis and Characterization of AgNPs, AgNPs-PEG, AgNPs-CIP, and AgNPs-PEG-CIP

The AgNPs, AgNPs-PEG, AgNPs-CIP, and AgNPs-PEG-CIP were prepared by adding SDS to control size and stability, with tri-sodium citrate dihydrate as a reducing agent, to produce the AgNPs. One of the most frequently employed methods to prepare AgNPs is by chemical reduction, due to the method’s high yield, low cost, and simplicity [22]. The PEG-400 polymer was added to improve the AgNPs’ stability and prevent their aggregation by decreasing the affinity of the composite to its own surface while retaining its high innate bactericidal activity. PEG is a hydrophilic polymer that improves stability in biological environments. In addition, PEG is a non-toxic, water-soluble, and biocompatible polymer that produces less toxicity than the NPs and also provides easy storage through loading and transportation [29,30].

**Figure 1:**
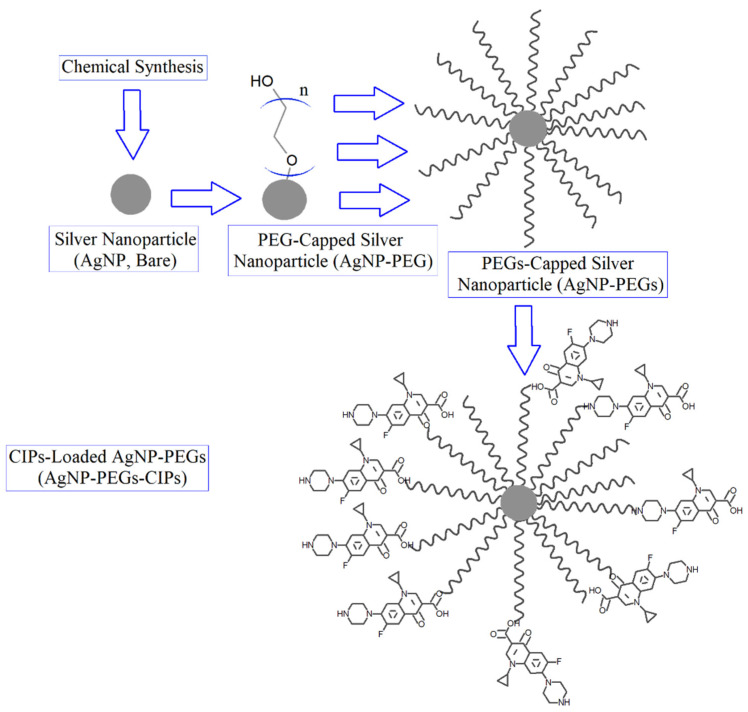
Schematic representation of the AgNP-PEG-CIP preparation and the molecular interaction sites (OH, NH, (C=O)-OH, and C=O) between the PEGs and the CIP molecules.

**Figure 2:**
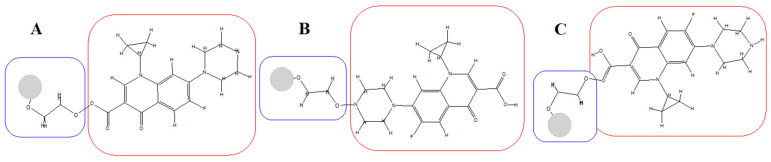
Chemical structure models of the AgNP-PEG-CIP (two methylene units,-CH_2_-CH_2_, of the AgNP-PEG, i.e., AgNP-O-(CH_2_-CH_2_)n-O-CIP interactions, showing OH, NH, and C=O sites); blue and red encased area represent PEG and CIP, respectively; the circle represents the AgNPs; (**A**) represents –(C=O)-OH-based CIP conjugation, (**B**) represents piperazinyl NH-based CIP conjugation, and (**C**) represents (HO-)-C=O-based CIP conjugation.

**Figure 3:**
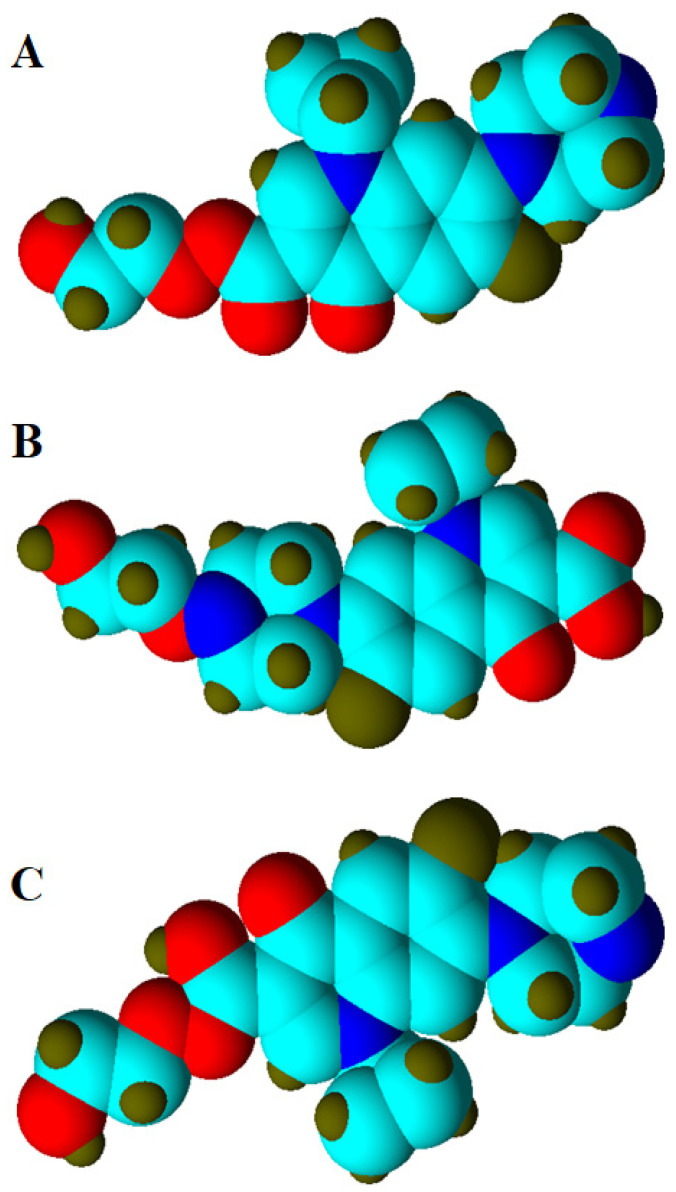
Space-filling chemical models of the AgNP-PEG-CIP (two methylene units,-CH_2_-CH_2_, of the AgNP-PEG-CIP, i.e., AgNP-O-(CH_2_-CH_2_)n-O-CIP)) interactions, showing compacted structures with OH, NH and C=O sites in close conjugations to the PEG unit: (**A**) –(C=O)-OH-based CIP conjugation, (**B**) piperazinyl NH-based CIP conjugation, (**C**) (HO-)-C=O-based CIP conjugation—the AgNPs are omitted from the space-filled models.

**Figure 4:**
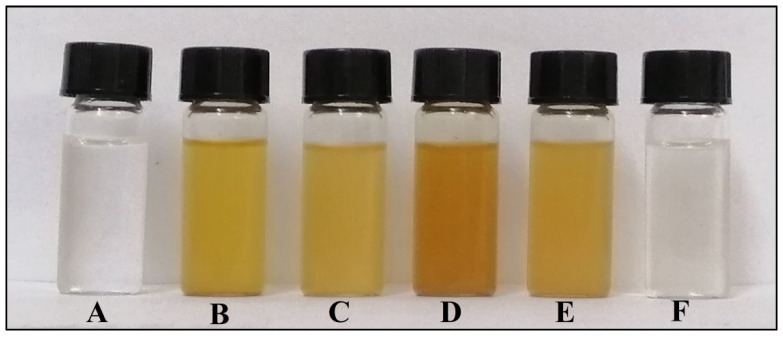
Chemically synthesized AgNPs and their conjugates: (**A**) AgNO_3_, (**B**) synthesized AgNPs, (**C**) AgNPs-PEG, (**D**) AgNPs-CIP, (**E**) AgNPs-PEG-CIP, and (**F**) Ciprofloxacin.

The loading of CIP onto the AgNPs was performed to enhance the activity of the antibiotic against the tested pathogenic bacteria as a powerful nano-antibiotic; changing color from yellow to orange was an important indicator of successful NPs-antibiotic conjugation [24]. The spectroscopic analysis has proven the antibacterial drug’s adhesion to the surface of AgNPs. A schematic representation of the process is outlined in Figure 1. The citrate-reduced AgNO_3_ yielded AgNPs that were coated with the PEGs; later, the CIP was loaded, as was confirmed through the observance of the corresponding UV-Vis spectroscopic absorption spectra and the FT-IR spectrum of these samples [31]. The observance of relevant peaks confirmed the presence of the functional groups OH, C=O, and NH, belonging to the PEG and CIP, and their conjugated presence in the AgNPs-PEG-CIP. An in silico representation suggested the interaction sites for the attachment of CIP’s C=O, (C=O)-OH, and NH with the other end of the PEG’s OH, whereby the former end of the PEG is attached to the AgNP, thus forming AgNP-PEG-CIP (Figure 2 and Figure 3). However, the first indicator of successful AgNP formation was the appearance of a yellow color in the previously colorless starting solution (Figure 4). The CIP-PEG attachments have provided a safe, non-reactive, and continuous release of the loaded drug and, most importantly, the conjugation seemed not to affect the structure activity relationship-based pharmacophore groups of the CIP molecules, of which the fluoro group is known to bind to the bacterial DNA gyrase, and the cyclopropyl ring is responsible for controlled pharmacokinetics and potency control. The piperzinyl group is also known to control the drug’s potency and contribute to the desired pharmacokinetics of the drug, while the carbonyl and the carboxyl groups are known for binding to the bacterial gyrase. The CIP molecules, being non-flat and wedge-shaped, unlike other fluoroquinolones molecules, have exhibited comparatively better antibacterial activity [32]. Furthermore, theoretical studies and parallel antibacterial activity experiments have also revealed the greater therapeutic effects of the AgNP-CIP conjugate when compared to CIP alone [33]. The AgNPs-PEG-CIP conjugate was also prepared as a strong, novel nano-antimicrobial agent with the greatest antibacterial effect. The CIP molecules attached to the hydroxyl (OH) group of the PEG provided high effectiveness of the PEG-conjugated AgNPs that were bound to CIP [30].

#### 3.1.1. Characterization by UV-Vis Spectrophotometry

**Figure 5:**
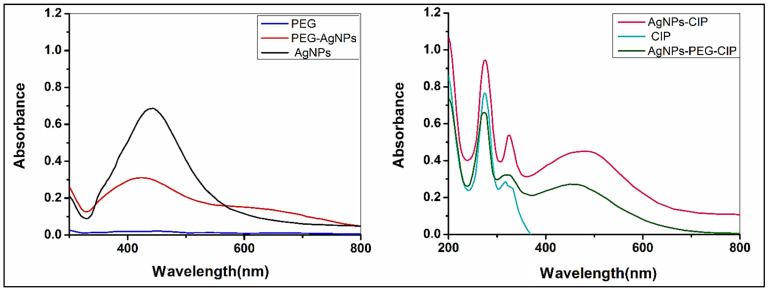
UV-Visible spectrum of AgNPs, CIP, PEG, and their conjugates.

Figure 5 shows the absorbances of AgNPs and their conjugates at between 200 and 800 nm. The peak at 438 nm is characteristic of AgNPs, while that at 426 nm indicated the formation of AgNPs-PEG, the difference in absorbance peaks is due to the increased particle size. These results were similar to those previously obtained by the authors of [22]. As for AgNPs-CIP, the characteristic SPR peak appeared at 477 nm; the increase was due to the color change, the surrounding chemical environment, and the size increase of the NPs because of the adsorption layer, thereupon representing a large shift toward the red spectrum following the addition of CIP, i.e., from 438 to 477 nm; the absorption peak of CIP alone was observed at 274 nm, as obtained previously [34]. The AgNPs-PEG-CIP conjugate showed an absorbance peak at 454 nm. The decreased absorbance compared to AgNPs-CIP may be due to the presence of PEG, indicating that the PEG and CIP were successfully conjugated to the AgNPs’ surface. Nevertheless, these particles were still nano-sized.

#### 3.1.2. FT-IR Analysis

**Figure 6:**
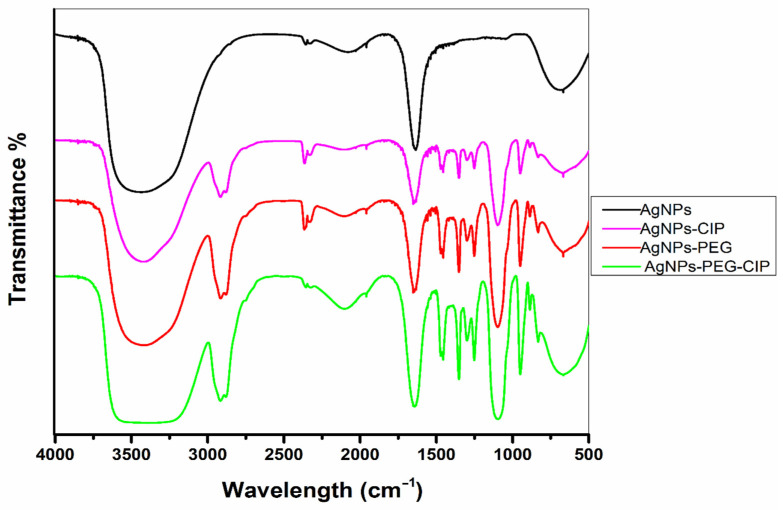
Fourier-transform infrared (FT-IR) spectra of AgNPs and their conjugates.

The AgNPs, AgNPs-PEG, AgNPs-CIP, and AgNPs-PEG-CIP samples were examined by FT-IR (Figure 6 and Table 1). The AgNPs showed transmittance peaks at 3435.56, 2354.57, 2080.76, 1958.82, 1635.80, 1455.50, 1046.57, and 667.44 cm^−1^. The peak at 3435.56 cm^−1^ was assigned to O-H (hydroxyl group) because a portion of O-H is inactive during the oxidation process, wherein some moisture is adsorbed onto the highly reactive surface of AgNPs [35]. As for the AgNPs-PEG, numerous peaks were observed: 3428.65, 2917.25, 2364.60, 1958.80, 1732.24, 1634.21, 1471.36, 1455.52, 1351.68, 1251.17, 1096.94, 950.15, and 667.27 cm^−1^. The interaction between AgNPs and PEG shifted the O-H hydroxyl group peak of AgNPs from 3455.56 cm^−1^ to 3428.65 cm^−1^, due to the conjugation with PEG, and a peak was detected at 2917.25 cm^−1^, corresponding to an aldehyde group stretching (C-H). The van der Waals interactions between the O-H bond with PEG and the partial positive charge on the surface of AgNPs resulted in the observed broad peaks [20,36].

The FT-IR analysis of AgNPs-CIP revealed peaks at 3434.62, 2919.39, 2356.04, 2089.68, 1635.59, 1456.07, 1352.56, 1383.56, 1251.73, 1094.57, 948.56, and 667.56 cm^−1^. The interaction between the C=O of the functionalized CIP and the AgNPs through a hydrogen (H) bond indicated their successful attachment. The transmittance peak at 1383.56 cm^−1^ was due to the amine (C-N) piperazine group of the CIP. In addition, the aryl C-H, and C-N stretching peaks were found at 2919.39 cm^−1^ and 1094.57 cm^−1^, respectively, in agreement with the previous observations [24]. The AgNPs-PEG-CIP conjugate showed IR transmittance peaks at 3413.61, 2914.81, 2331.60, 2366.04, 2103.46, 1958.81, 1634.15, 1455.59, 1351.84, 1299.13, 1251.00, 1098.04, 950.17, 886.62, 832.67, and 667.37 cm^−1^. The O-H group peak shifted from 3428.65 cm^−1^ to 3413.61 cm^−1^ in AgNPs-PEG-CIP and the peak showed increased intensity, indicating the interactions of AgNPs, PEG, and CIP [30].

#### 3.1.3. XRD Analysis

**Figure 7:**
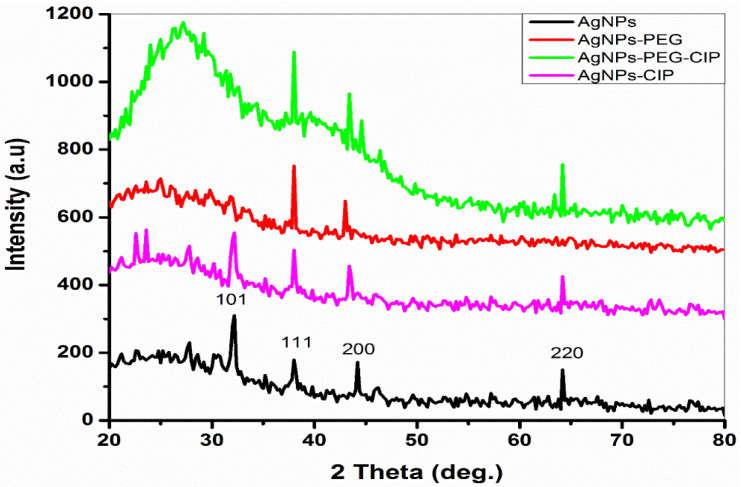
X-ray diffraction pattern of AgNPs and their conjugates.

The XRD pattern of AgNPs at angle 2θ showed four peaks at 32.12°, 38.04°, 46.21°, and 64.18°, corresponding to the (101), (111), (200), and (220) planes, respectively, when compared with the Joint Committee on Powder Diffraction Standards (JCPDS), File no. 04-0783 and 84-0713 (Figure 7). Four peaks were also detected for AgNPs-CIP at angle 2θ: 32.22°, 38.04°, 44.42°, and 64.24°. These peaks corresponded to planes (101), (111), (200), and (220), respectively. In the case of AgNPs-PEG, only two angle 2θ peaks were found, at 38.02° and 44.12°, which corresponded to planes (111) and (200), respectively. The XRD pattern of AgNPs-PEG-CIP at angle 2θ consisted of three peaks at 38.41°, 44.62°, and 64.43°, corresponding to the (111), (200), and (220) planes, respectively. These findings are consistent with previous reports [37,38,39].

The crystallite size (D) of AgNPs and their conjugates were calculated using the Scherrer equation (Equation (1)) for the highest peak in the XRD pattern, using K = 0.9 (D = 0.9λ/βcosθ). The D of AgNPs was ~36.3 nm, according to the expansion of the (101) reflection, while the D of AgNPs-PEG was ~52.5 nm, according to the expansion of the (111) reflection. According to this reflection, the D of AgNPs-PEG-CIP was ~67.4 nm. These sizes were similar to those observed in the SEM analysis. Determining the size of NPs provided information on their effectiveness and behavior as an antibacterial agent. The smaller the NP sizes, the higher the level of activity they possess. The FT-IR allowed us to identify the components of the materials and also indicated their molecular associations, together with the X-ray analysis, which confirmed their physical state of crystalline structure; thus, it was possible to identify the extent of crystal formation in the AgNPs using this technique [40].

#### 3.1.4. Size and Zeta Potential Measurements

**Figure 8:**
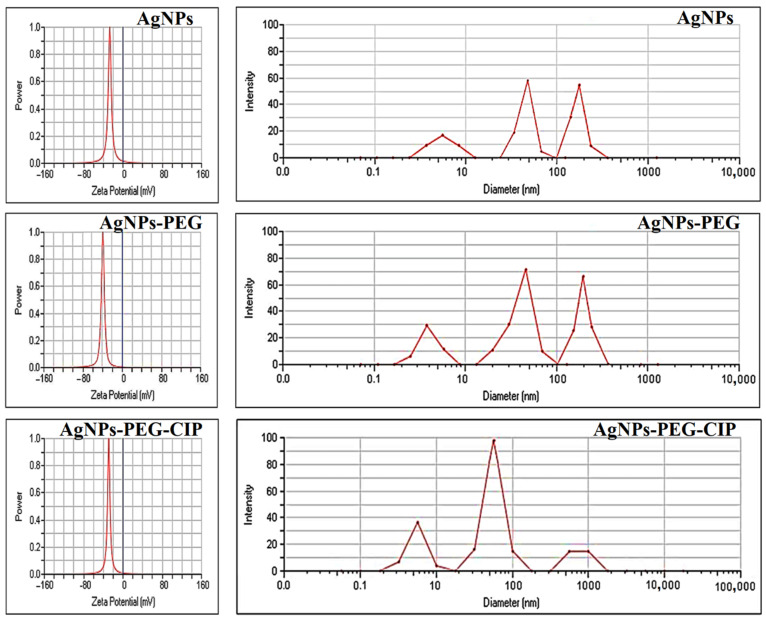
Zeta potentials and dynamic light scattering analyses of AgNPs, AgNPs-PEG, and AgNPs-PEG-CIP.

The DLS results (Figure 8) showed that the mean particle size of the synthesized AgNPs was 57.9 nm, with a polydispersity index (PDI) of 0.334, confirming the nanoscale size of the AgNPs. As for the AgNPs-PEG, the DLS reading was 72.63 nm, with a PDI value of 0.277, while the DLS of the AgNPs-PEG-CIP was 97.31 nm with a PDI value of 0.317, indicating a remarkable increase in particle sizes due to the layering with the PEG and CIP conjugations [21,41]. The PDI is an important index for NP size distribution [42]. The decreasing PDI from AgNPs to AgNPs-PEG indicated the generation of a large number of monodispersed NPs due to the high concentration of PEG, which prevented the aggregation of NPs [43].

The zeta potential was used to test the stability and surface charge of the AgNPs and their conjugates. Highly unstable, relatively unstable, moderately stable, and highly stable colloids presented zeta potential ranges of 0–10 mV, 10–20 mV, 20–30 mV, and >30 mV, respectively [36]. The zeta potential of the AgNPs was −26.84 mV at room temperature, and these NPs were considered moderately stable. The zeta potential of AgNPs-PEG was−37.66 mV, corresponding to a higher negative surface charge than that of the AgNPs alone. The increased zeta potential value with the presence of PEG indicated considerable electrostatic repulsion and implied that this form was the most stable (in deionized water), which is in agreement with the notion of PEG working as a stabilizer, thereby limiting the mobility of the Ag ions and preventing the NPs’ agglomeration during the nanoparticle preparation reaction, whereas the long hydrophilic chains of the PEG were associated with water molecules, and were responsible for preventing the agglomeration, thus increasing the number of freely suspending AgNPs [20]. The AgNPs-PEG-CIP presented a zeta potential of −29.46 mV, which was lower than that of the AgNPs-PEG, implying that the solution had long-term stability. The negative value reinforced the particle repulsion behavior and, as a result, increased the stability of the AgNPs-PEG-CIP. Moreover, the negatively charged surfaces aided in preventing the NPs from aggregating, and also contributed to managing the NPs’ shape and size [44].

#### 3.1.5. FE-SEM Analysis

**Figure 9:**
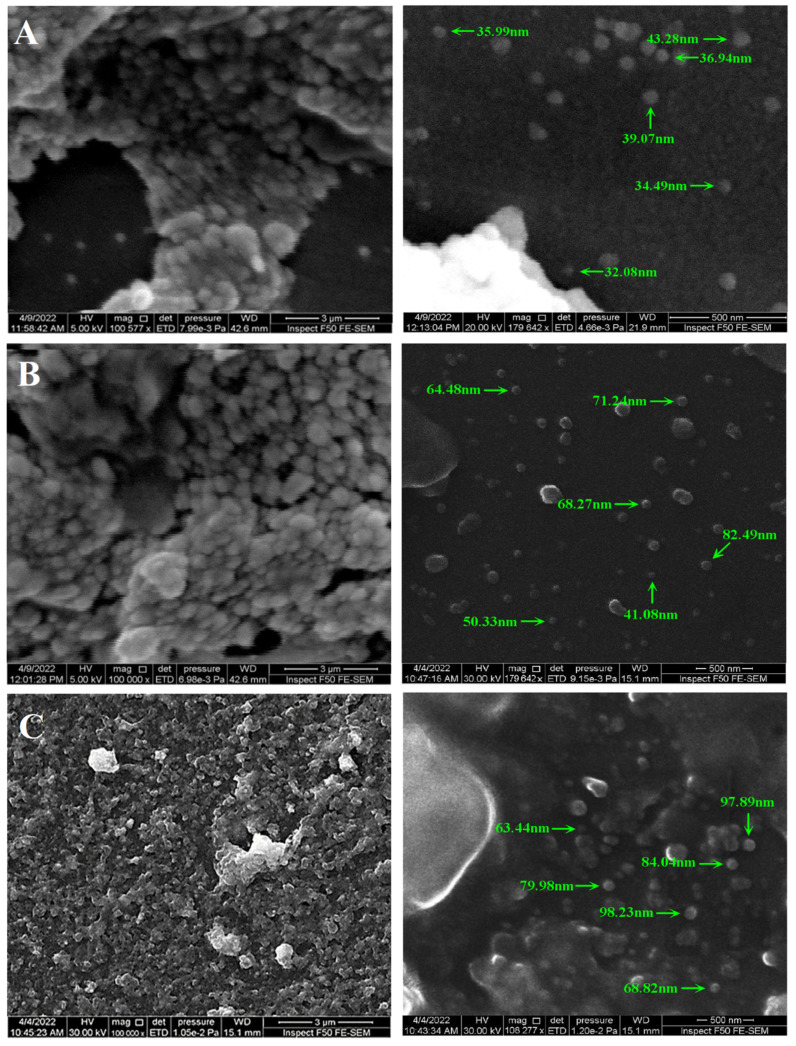
SEM images (left panels; scale bar 3 µm) and particle size measurements (right panels; scale bar 500 nm). (**A**) AgNPs, (**B**) AgNPs-PEG, and (**C**) AgNPs-PEG-CIP.

The size, surface morphology, and uniformity of NPs were evaluated using FE-SEM. The technique allowed acquiring both the qualitative and quantitative data, as well as other details related to the NPs’ morphology and size [45]. ImageJ software (Java 1.8.0, Gaithersburg, MD, USA) was utilized to determine the diameter of the particles that were synthesized at the nanometer scale. Figure 9A shows the majority of the AgNPs as spherical-shaped, with a smooth surface, and exhibited particle agglomeration and aggregation. The structures were 32.08 nm to 43.28 nm in size, which was in agreement with the XRD observations. The AgNPs-PEG showed an increased size range (41.08 to 82.49 nm, Figure 9B), which was expected due to the PEG coating. The effect of PEG on preventing particle aggregation was also evident, as the AgNPs-PEG particles appeared to be dispersed. The SEM images of the AgNPs-PEG-CIP revealed particles from 63.44 to 98.28 nm in size, which was due to both the PEG and CIP coatings; however, a slight aggregation of the particles was noticed (Figure 6C). Nonetheless, the size ranges of the NPs observed through the SEM measurements were significantly lower than those obtained using the DLS technique. Observations similar to these were also reported in a previous study [46,47]. These differences might be due to the fact that SEM images generally reflect the metallic core at the center of the particles [48], while the DLS-based measurements depended on the mean hydrodynamic diameter. In addition, the small aggregates in the nanosuspensions may also have been measured using the DLS technique, affecting the size distribution of the prepared NPs [46].

**Figure 10:**
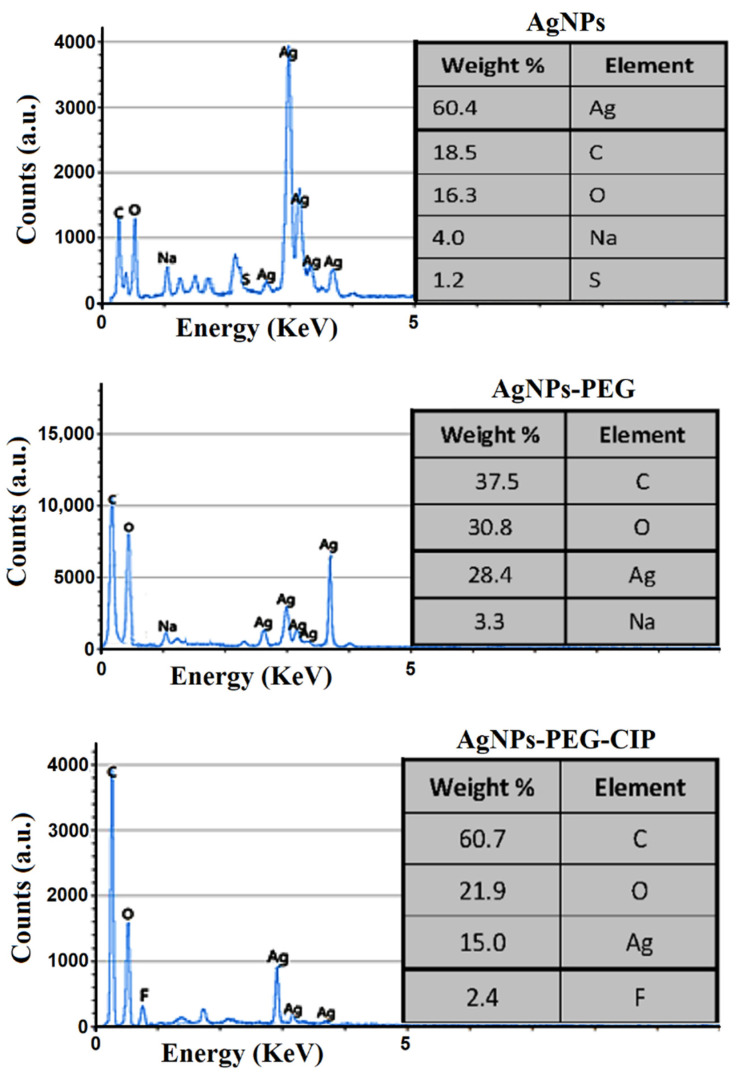
EDX spectra for AgNPs, AgNPs-PEG, and AgNPs-PEG-CIP.

The energy-dispersive X-ray (EDX) analysis revealed that the weight percentage of Ag in the AgNPs was 60.4% of the total sample components, with only small percentages of carbon (C), oxygen (O), sulfur (S), and sodium (Na) present; these were the component parts of the chemicals used for the AgNPs synthesis (Figure 10). For AgNPs-PEG, the EDX analysis showed a lower percentage of Ag (28.4%), and higher percentages of C (37.5%) and O (30.8%), in contrast to the AgNPs, due to the presence of PEG [49]; a small percentage of Na was also found in these samples. The AgNPs-PEG-CIP conjugate contained an even lower percentage of Ag (15%) but higher percentages of C and O, due to the addition of the coated PEG polymer and the antibiotic conjugates. In addition, a small percentage of fluorine (F), related to the presence of CIP, was also found.

#### 3.1.6. TEM Analysis

**Figure 11:**
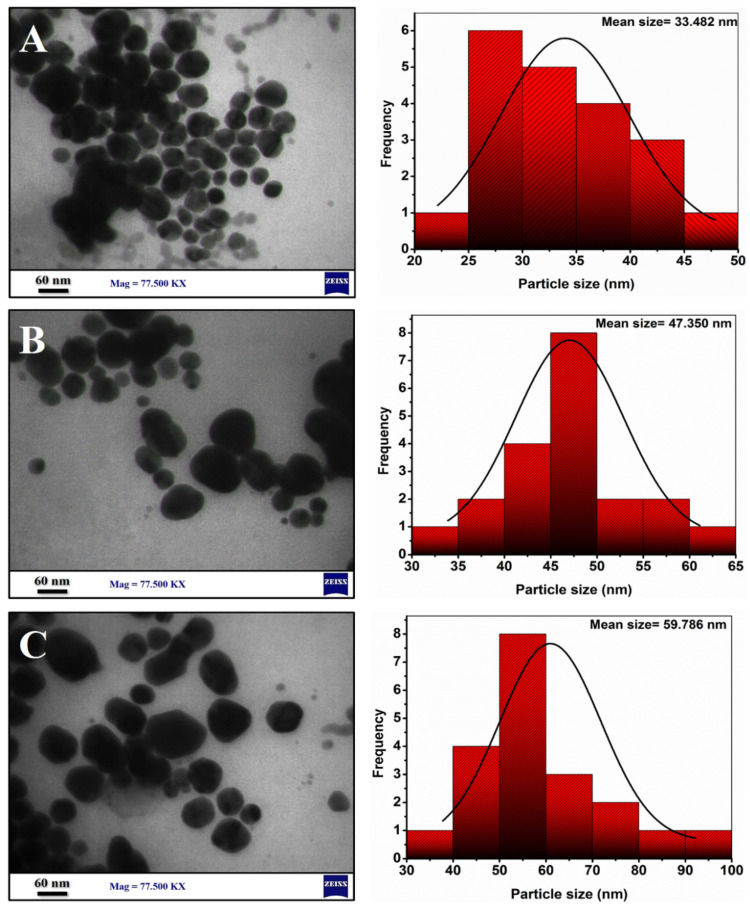
TEM images of AgNPs and their conjugates: (**A**) AgNPs, (**B**) AgNPs-PEG, and (**C**) AgNPs-PEG-CIP.

To analyze the morphology and size distributions of the synthesized AgNPs, AgNPs-PEG, and AgNPs-PEG-CIP, TEM analysis was performed using 77.500 kx magnification power. The magnified image results demonstrated that the majority of NPs have spherical shapes, as seen in Figure 11, and the histogram of size distribution exhibited a mean size for AgNPs of 33.48 nm, of 47.35 nm for the AgNPs-PEG, and of 59.78 nm for the AgNPs-PEG-CIP, as calculated using the ImageJ software. Following PEG addition, the NPs showed less agglomeration because of PEG’s ability to prevent the particles from aggregating [50]. The PEG molecules that were bound to the AgNPs increased the steric effects, bulkiness, and distances between the nanoparticles. The hydrophilicity of the NPs also increased through the formation of hydrogen bonds with the solvent, thus preventing the NPs from aggregating further [51]. The AgNPs-PEG-CIP particles are predominantly spherical in shape, with some being triangular and some of them being rod-shaped. These particles, AgNPs-PEG-CIP, were observed to be larger in size than the AgNPs and AgNPs-PEG. However, the particle sizes were smaller than the size revealed by the DLS analysis, which apparently is due to the dehydration of the sample that occurred during the TEM analysis and processing of the material [52,53].

### 3.2. Antioxidant Activity

**Figure 12:**
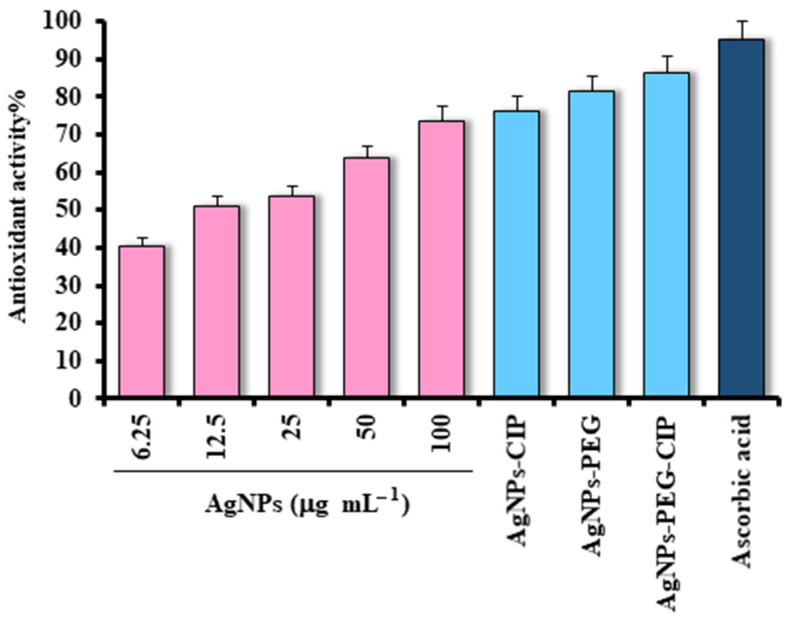
Antioxidant activity of AgNPs, AgNPs-CIP, AgNPs-PEG, and AgNPs-PEG-CIP using the DPPH assay method. Ascorbic acid was used as the positive control.

DPPH, a free radical that is stable at room temperature, displays a dark violet color when dissolved in organic solvents and shows an absorption wavelength of 517 nm. When AgNPs were present in the analysis, the DPPH stability was found to have decreased, and the violet color turned yellow due to the presence of phenolic OH groups [54]. The assays conducted for AgNPs, AgNPs-PEG, AgNPs-CIP, and AgNPs-PEG-CIP showed that DPPH was scavenged proportionally to the concentrations of the AgNPs, i.e., at concentrations of 6.50, 12.5, 25, 50, and 100 μg mL^−1^ of AgNPs, the DPPH free radicals’ scavenging capacities were 40.45%, 50.98%, 53.66%, 63.67%, and 73.64%, respectively (Figure 12). The antioxidant activities of the AgNPs-CIP and AgNPs-PEG were at 76.24% and 81.44%, respectively. The highest scavenging activity was obtained for AgNPs-PEG- CIP at 86.34%. However, this was still lower than the antioxidant activity of the ascorbic acid, the positive control standard that was used, which showed strong antioxidant activity [55]. The antioxidant activities of the AgNPs-CIP, AgNPs-PEG, and AgNPs-PEG-CIP were measured at 100 μg mL^−1^ of concentration.

### 3.3. Antibacterial Activity

**Figure 13:**
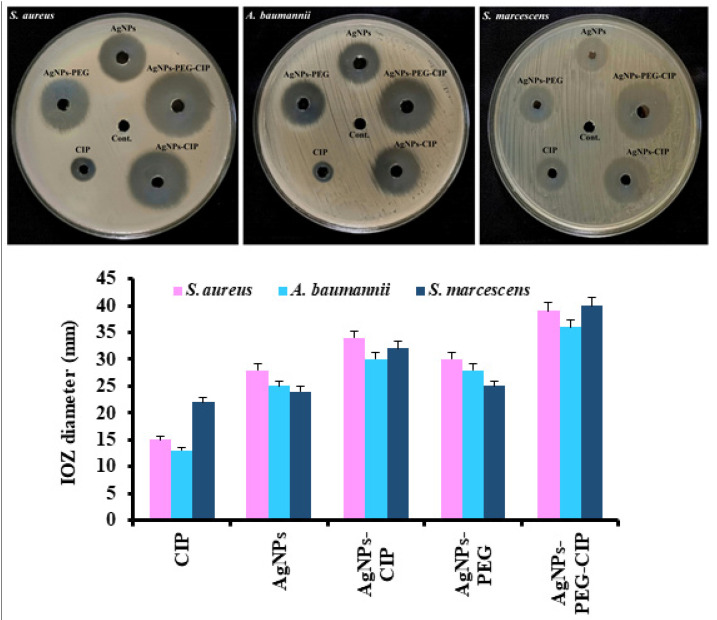
The synergistic effects of the AgNPs-PEG, AgNPs-CIP, and AgNPs-PEG-CIP on the growth of pathogenic bacteria, compared to CIP and to AgNPs alone.

The synergistic antibacterial effects of the AgNPs-CIP, AgNPs-PEG, and AgNPs-PEG-CIP on the growth of bacterial isolates were compared to that of the AgNPs and of CIP alone. These results showed the increased effects of CIP on all tested bacteria after conjugation with AgNPs. The best effect was observed for the AgNPs-PEG-CIP, as shown in Figure 13. The synergistic effects of the AgNPs and CIP were demonstrated by the increased diameter of the inhibition zones of the bacterial strains that were resistant to CIP. The inhibition zone of AgNPs alone was 29.33 ± 1.80 mm, 26.66 ± 1.52 mm, and 24.66 ± 2.08 mm, while that of CIP alone was 16.00 ± 2.12 mm, 13.33 ± 0.57 mm, and 23.33 ± 1.54 mm for *S. aureus*, *A. baumannii*, and *S. marcescens*, respectively. In the case of the AgNPs-CIP, the largest inhibition area (35.33 ± 1.52 mm) was obtained for *S. aureus*, followed by 32.66 ± 1.15 mm for *S. marcescens* and 31.00 ± 1.27 mm for *A. baumannii*. The sensitivity of *S. marcescens* to CIP increased after the antibiotic was loaded onto the AgNPs, as the inhibition area increased from 22 mm with CIP alone to ~32 mm for the AgNPs-CIP, which interfered with the bacterial cell wall causing the cell lysis. Conjugation of the AgNPs with CIP also facilitated antibiotic entry into the bacterial cells, as DNA gyrase enzyme and topoisomerase IV activities have been reported to be blocked, thereby inhibiting the cell division and eventually leading to bacterial cell death [56,57]. When the MNPs are mixed with antibiotics, the amount of drug required is low, which also minimizes its toxicity and the risk of developing bacterial resistance [58]. The AgNPs-CIP showed higher antibacterial activity against Gram-positive bacteria than against Gram-negative bacteria. This is due to differences in the molecular composition of the cell wall of these strains of bacteria [59]. Gram-positive bacteria have a rigid and thick peptidoglycan layer that is absent in Gram-negative bacteria, while the latter have a 10-nm thick lipopolysaccharide (LPS) layer covering the peptidoglycan outer layer, thus hindering the NPs’ access to the cell wall [54].

The AgNPs-PEG-CIP showed strong antibacterial effects against the growths of all the tested pathogenic bacteria, with the inhibition zone diameters reaching ~36 mm for *A. baumannii*, ~39 mm for *S. aureus*, and ~40 mm for *S. marcescens*. Therefore, the sensitivity of these bacteria toward CIP significantly increased after PEG-400 addition, which was also supported by the slightly increased inhibition zones of the AgNPs-PEG, compared to the AgNPs-CIP (Figure 13). Given the strong hydrophilic nature of the PEG-400, a large amount of water can be taken up by the PEG units and the water that is present can then be removed from the bacterial cells inhibiting their growth, as the bacteria require specific quantities of water to grow. Encapsulation is another important aspect contributing to the stronger inhibitory effects of the AgNPs-PEG, because the coated polymeric layer can reduce the surface energy of the NPs and the surface modification helps to inhibit agglomeration, resulting in long-term stability [60]. As PEG-AgNPs had a longer PEG chain surrounding them, they demonstrated better antibacterial action; the inhibition area of the AgNPs-PEG ranged from ~2 to ~3 mm.

The AgNPs-PEG-CIP acted as a platform for drug delivery. The AgNPs attached to the PEG showed enhanced biocompatibility and the PEG worked as a linker to the CIP and to the surface of the AgNPs on the other end [21]. In fact, the PEG polymer alone has been employed as a drug delivery system due to its greater biocompatibility and non-toxicity, compared to several other polymers [58]. The significant increases in the inhibition zones compared with the AgNPs-PEG indicated that the improved antibacterial activity resulted from the slow release of the loaded CIP molecules from the AgNPs-PEG-CIP nanoconjugate [33]. The outermost layer of the CIP will dissolve quickly, but the interior core will need to be hydrated before their dissolution. Thus, the initial size of the anhydrous CIP crystal (the specific surface area) greatly affected its solubility. The PEG can decrease the size of the CIP nanoconjugates, hence increasing their solubility, enhancing CIP release in a slow fashion that is dependent upon achieving hydration. As a result, the direct effects of the PEG on the CIP crystals represent another crucial factor that promotes CIP release from the AgNPs-PEG-CIP [58]. The observed strong antibacterial effects of the AgNPs-PEG-CIP are therefore due to the increased release of the CIP molecules. The antibacterial activity of the synthesized AgNPs is highly dependent on their physicochemical properties, such as their chemical composition, size, and shapes. A study by Mohsen and his co-workers reported that the prism-shaped AgNPs exhibited significant bacterial inhibition against Gram-negative (*E. coli*) and Gram-positive (*S. aureus*) bacterial strains, compared to the spherical-shaped AgNPs. Their efficient antibacterial properties, due to the vertexes and the numerous sharp edges of the nano-prisms, facilitated the penetration of the AgNPs nanoformulation across and into the cell walls, causing the destruction of the bacterial cell wall [24].

### 3.4. Antibiofilm Activity

The effects of bare AgNPs and AgNPs-PEG, and AgNPs and AgNPs-PEG-CIP on biofilm formations under laboratory conditions were studied by observing the bindings of the crystal violet (CV) formula to the adherent cells, which directly reflected the effective ability of these formulations to inhibit biofilm formation. The results of the 0.1% CV staining assay showed that all the tested bacteria were able to produce biofilm and the AgNPs and AgNP-based formulation inhibited biofilm production, which explained the high resistance rate of most of the formulations, i.e., AgNPs, AgNPs-PEG, and AgNPs-PEG-CIP, as antibiotics. However, when treated with different concentrations of AgNPs and their conjugates, it was evident that the nanoconjugates easily eliminated the biofilm. According to the results, the strongest inhibition effect was obtained for AgNPs-PEG-CIP nanoconjugate. The positive control tube was found to consist of a dense biofilm (+++), while the negative control tubes failed to form a biofilm (−).

**Figure 14:**
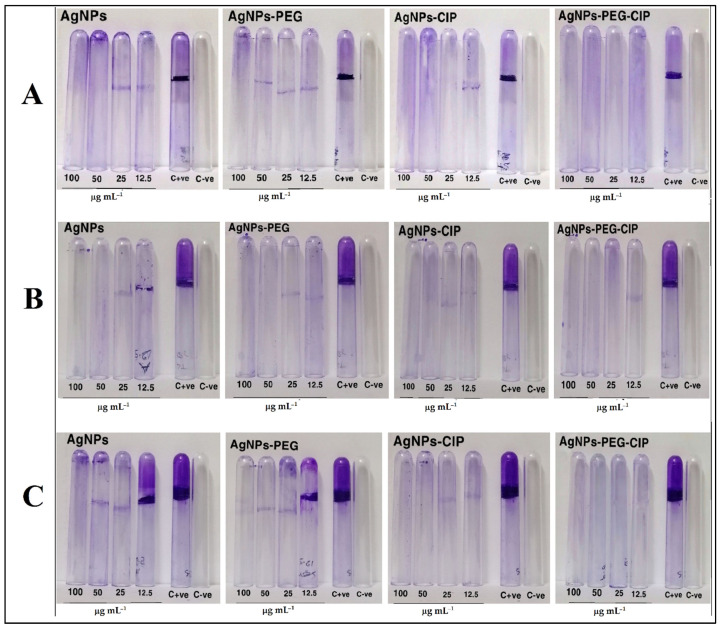
The anti-biofilm effects of AgNPs and their conjugates at 12.5, 25, 50, and 100 μg mL^−1^ concentrations by (**A**) *S. aureus*, (**B**) *A. baumannii*, and (**C**) *S. marcescens.*

Table 2 summarizes the results for all bacterial strains treated with AgNPs and their different conjugates at different concentrations (Figure 14). AgNPs-CIP and AgNPs-PEG-CIP at 50 μg mL^−1^ inhibited the biofilm formation (−), while AgNPs only, or AgNPs-PEG, allowed the formation of a weak biofilm (+) at 50 μg mL^−1^ (except for *S. aureus*, where no biofilm appeared), and moderate biofilm formation (++) at 25 μg mL^−1^. Two hypotheses have been put forward to explain these effects: (i) Ag ions may weaken the biofilm formation in bacteria by moving into the bacterial cell and interfering with the proteins and enzymes required for microbial adhesion, which results in decreased biofilm formation [61]; (ii) the AgNPs inhibit biofilm formation by inhibiting the formation of exogenous polysaccharides (exopolysaccharides). The NPs inhibit the biofilm formation process by penetrating the water channels (aquapores) that transport water and nutrients through the layers of polysaccharides present on the bacterial cell wall [62].

### 3.5. Determination of MIC and MBC

**Figure 15:**
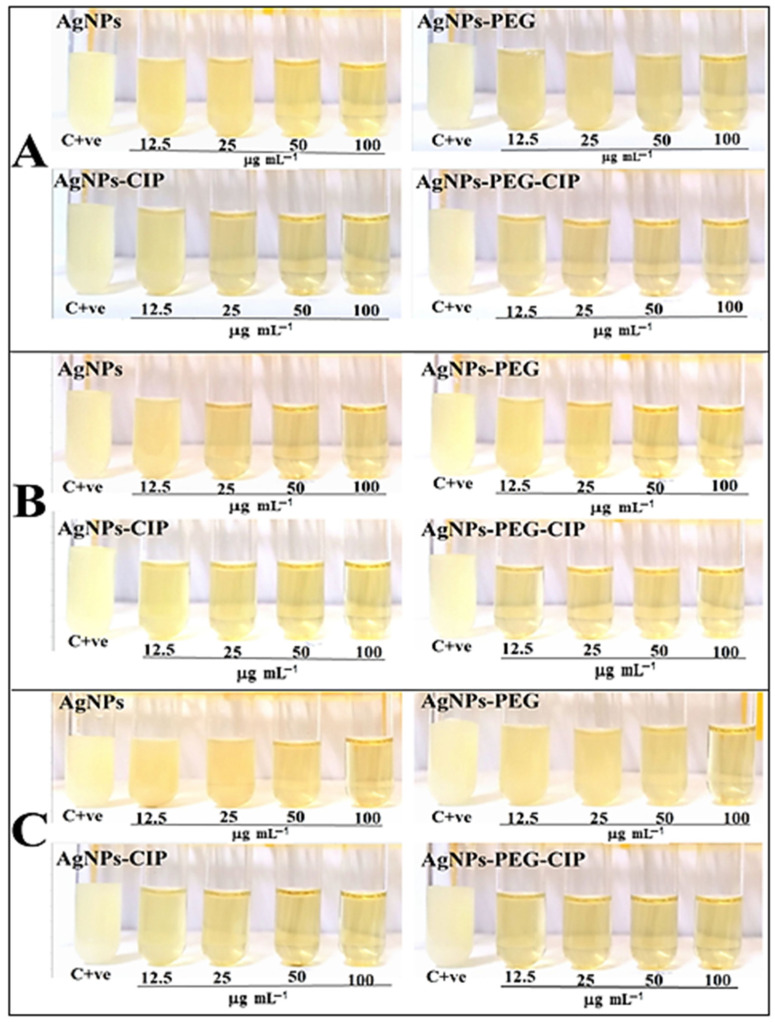
Minimum inhibitory concentrations (MIC) and minimum bactericidal concentrations (MBC) of the AgNPs and their conjugates against pathogenic bacteria: (**A**) *S. aureus*, (**B**) *A. baumannii*, and (**C**) *S. marcescens.*

Using turbidity assays, the influence of AgNPs, AgNPs-PEG, AgNPs-CIP, and AgNPs-PEG-CIP were determined according to the growth of the bacterial isolates employed in the present study, using liquid culture media. The MIC of AgNPs and AgNPs-PEG for *S. aureus* was at 25 μg mL^−1^, while that for AgNPs-CIP and AgNPs-PEG-CIP was at 12.5 μg mL^−1^. For *A. baumannii*, the MIC of AgNPs and AgNPs-PEG were at 50 μg mL^−1^ and 25 μg mL^−1^ for AgNPs-CIP, while for *S. marcescens*, the MIC was 50 μg mL^−1^ for AgNPs and AgNPs-PEG and 12.5 μg mL^−1^ for AgNPs-CIP, respectively. These results indicate that the MIC of AgNPs and AgNPs-PEG for *S. aureus* were lower than for the other tested bacteria (Figure 15). Among all the tested bacteria, the lowest MIC value was obtained for the AgNPs-PEG-CIP, which showed high antibacterial activity with a significant decrease in the MIC, compared to CIP and AgNPs alone. The MIC value of AgNPs-CIP decreased compared to that of the bare AgNPs and the coated AgNPs, due to the synergistic effects of the PEG and AgNPs, as also observed in a previous study [57]. The presence and absence of the bacterial growth on solid culture media were used to calculate the MBC, which is the designated concentration at which the tested bacteria were killed, and the test tubes appeared clear [60]. The MBC values were higher than the MIC values. Accordingly, the MBC of the AgNPs alone and for AgNPs-PEG for *A. baumannii* and *S. marcescens* were at 100 μg mL^−1^ and at 50 μg mL^−1^ for *S. aureus.* As for the AgNPs-CIP, the MBC was at 50 μg mL^−1^ for *A. baumannii* and at 25 μg mL^−1^ for *S. marcescens* and *S. aureus*. The MBC values of the AgNPs-PEG-CIP showed strong effects in inhibiting bacterial growth, compared to the AgNPs and CIP alone and the AgNPs-CIP. The MBC values for *S. aureus*, *A. baumannii*, and *S. marcescens* were at 25, 50, and 12.5 μg mL^−1^, respectively. In general, the major mechanism of AgNPs actions in an aqueous microenvironment was that the silver nanoparticles were continuously released as silver ions; the reactive oxygen species (ROS), together with free radicals, were produced by these silver nanoparticles, which damaged the bacterial cell wall and blocked the respiratory enzymes. The value of the MBC was affected by the nature of the cell wall, the bacterial production of certain enzymes, and the concentrations of the bacterial suspension [63].

## 4. Conclusions

The findings of the present study revealed that the loading of the ciprofloxacin fluoroquinolone antibiotic onto chemically synthesized AgNPs provided an effective formulation against pathogenic bacterial isolates, i.e., *S. aureus*, *A. baumannii*, and *S. marcescens*, compared to the bare silver nanoparticles and molecularly free ciprofloxacin. The antibacterial activities were confirmed by their resistance to these studied pathogens. Strong antibacterial nano-conjugates were obtained when PEG-400 was used as a linker and stabilizer to attach CIP and prepare the AgNPs-PEG-CIP. The AgNPs-PEG also provided stability and zeta potential control to the prepared nanocolloidal suspension, and decreased the aggregation of the AgNPs, although not very significantly. The chemical reduction method used to synthesize the AgNPs provided relatively stable NPs, and the AgNPs and their subsequently prepared conjugates were characterized by their physicochemical properties in analyses (shape, size, and chemical compositions). The AgNPs-PEG-CIP was obtained as a novel drug delivery nano-conjugate that showed excellent inhibition of bacterial growth, anti-biofilm activity, and strong antioxidant activity. Overall, the results confirmed that AgNPs capped with PEG acted as a suitable platform for delivering conjugated ciprofloxacin, owing to their stability and the non-aggregative nature of the prepared nanosuspension in the final formulation. The effects of the intermediate product, AgNPs-PEG, also showed slight antibacterial effects due to its hydrophilic characteristics. AgNPs and their conjugates’ antioxidant activity in DPPH assays also confirmed their potency levels for the produced ROS quenching. ROS levels are a crucial factor affecting bacterial growth by damaging the bacterial cell wall and deactivating certain bacterial enzymes. The simplicity of the synthetic process and the remarkable biological activity of these antimicrobial nano-conjugates present the potential to provide a wide range of opportunities for their adoption as an effective and successful antibiotic in clinical therapeutics, as part of contemporary nanomedicine.

## Figures and Tables

**Table 1:** Functional groups in AgNPs and their PEG and CIP nano-conjugates.

Bonding Types	Functional Groups	Products and Wavelengths (cm^−1^)			
		AgNPs	AgNPs-PEG	AgNPs-CIP	AgNPs-PEG-CIP
O-H	Hydroxyl	3455.56	3428.65	3434.62	3413.61
C-H	Aryl	-	-	2919.39	2914.81
C=O	Carbonyl	1635.80	1634.21	1635.59	1634.15
N-H	Amine	1046.57	1096.94	1094.57	1098.04
C=C	Alkene	667.44	667.27	667.56	667.37

**Table 2:** Effect of AgNPs and their conjugates on the biofilm produced by the tested bacteria.

Bacteria	Concentrations of Silver Nanoparticles (μg mL^−1^)															
	AgNPs				AgNPs-PEG				AgNPs-CIP				AgNPs-PEG-CIP			
	12.5	25	50	100	12.5	25	50	100	12.5	25	50	100	12.5	25	50	100
***S. aureus***	**+**	**+**	**−**	**−**	**+**	**+**	**+**	**−**	**+**	**−**	**−**	**−**	**−**	**−**	**−**	**−**
***A. baumannii***	**++**	**+**	**−**	**−**	**++**	**+**	**−**	**−**	**+**	**+**	**−**	**−**	**+**	**−**	**−**	**−**
***S. marcescens***	**+++**	**++**	**+**	**−**	**+++**	**++**	**+**	**−**	**+**	**+**	**−**	**−**	**−**	**−**	**−**	**−**

**Note**: (+) Weak biofilm, (++) moderate biofilm, (+++) dense biofilm, and (−) no biofilm.

## Data Availability

All the data were provided in the manuscript.

## List of Abbreviations

AgNPs	Silver Nanoparticles
CIP	Ciprofloxacin
CV	Crystal Violet Stain
DPPH	1,1-Diphenyl-2-picryl-hydrazyl
MBC	Minimum Bactericidal Concentration
MIC	Minimum Inhibitory Concentration
PBS	Phosphate Buffer Saline
PEG	Polyethylene Glycol
TSB	Tryptic Soy Broth
